# Implicit learning of social information in contextual cueing

**DOI:** 10.1186/s41235-026-00736-8

**Published:** 2026-06-23

**Authors:** Lijeong Hong, Min-Shik Kim

**Affiliations:** Department of Psychology, Yonsei University, 50 Yonsei-Ro Seodaemun-Gu, Seoul, 03722 Korea

**Keywords:** Implicit learning, Object contextual cueing, Visual search, Social perception, Attention, Ensemble coding

## Abstract

Human attention is often guided by contextual cues that signal where or how to focus in complex environments. Research on object contextual cueing has largely emphasized spatial regularities, but it remains unclear whether socially meaningful information can also provide such guidance. Across three experiments using face stimuli, we examined whether social cues can serve as contextual cues independently of spatial arrangements. In all experiments, participants searched for a target face among distractor faces, with spatial configurations fully randomized on every trial, and contextual information was defined by different types of social mappings. In Experiment 1, the cue was defined by consistent associations between a target identity and a specific set of distractor identities. In Experiment 2, the cue was the overall mood, quantified as the ratio of angry to happy faces. In Experiment 3, the cue was relational information, specifically facing-direction patterns within pairs of profile-view faces. Performance under Consistent Mapping conditions was compared with Variable Mapping conditions, in which these social regularities changed across trials. Bayesian analyses revealed reliable contextual cueing effects across all three experiments, with faster responses under Consistent than Variable Mapping conditions. The effect was strongest in Experiment 2, suggesting that global social cues may be encoded more efficiently than local relational cues. Post-experiment awareness tests indicated minimal explicit knowledge and no reliable association between awareness measures and contextual cueing. Together, these findings suggest that socially meaningful relational structure can provide stable predictive information that supports object-based contextual learning even when spatial regularities are absent.

**Supplementary Information:**

The online version contains supplementary material available at https://doi.org/10.1186/s41235-026-00736-8.

## Significance statement

Interpreting complex social environments requires the rapid extraction of meaningful information from dynamic scenes. In everyday contexts—such as classrooms, meetings, or social gatherings—people often rely on subtle cues about relationships, group mood, or who is attending to whom to efficiently locate and interpret relevant information. Classic contextual cueing research has shown that observers implicitly learn repeated spatial arrangements, using them to guide attention during visual search. The present study extends this framework to social contexts. Across three controlled experiments, we demonstrate that people implicitly learn abstract social regularities—such as identity associations, the overall mood of a group, or facing-direction patterns—and subsequently use these cues to guide attention, even when they are task-irrelevant and when spatial regularities are entirely removed. These findings suggest that socially meaningful relational structure can provide a predictive scaffold that stabilizes contextual learning, enabling attention to be guided by structured social information even in the absence of spatial consistency. Understanding how people automatically extract and use social structure has implications for research on social attention, atypical social processing, and the development of artificial systems that interpret social environments.

People naturally extract and learn statistical associations between entities that frequently co-occur in daily life. For instance, we form expectations about familiar pairs of characters in media, coworkers who routinely interact, or everyday objects that typically appear together. Such associations are acquired effortlessly through visual experience, reflecting sensitivity to covariation among entities.

This sensitivity to co-occurrence has been most prominently demonstrated in the contextual cueing paradigm (Chun & Jiang, 1998). In their classic study, participants searched for a target letter among distractors, with some spatial arrangements repeating throughout the experiment while others remained novel. Over time, participants responded faster to repeated than to novel displays, indicating that they had learned the spatial context and used it to guide attention. The effect develops implicitly, even when participants cannot report the repeated configurations, suggesting an automatic mechanism through which environmental structure facilitates behavior.

Contextual cueing, however, is not limited to spatial regularities (Sisk et al., 2019). Semantically related distractor words (Goujon et al., 2009), object identity pairings (Chun & Jiang, 1999), motion patterns (Chun & Jiang, 1999), and even cross-modal cues (Kawahara, 2007) can support contextual learning. In some cases, stable target–distractor identity relations facilitate search even when spatial arrangements vary randomly from trial to trial. Yet these findings coexist with evidence showing important limits, as attempts to reproduce object-based contextual cueing in isolation have yielded inconsistent results. For example, Makovski (2016) showed that repeating object identities without stable spatial configurations often fails to produce contextual cueing, and further demonstrated that separating semantic meaning from visual similarity can disrupt performance (Makovski, 2018). Similarly, Chen et al. (2024) reported that contextual cueing did not reliably emerge when distractor identity information was manipulated independently of spatial regularities. More recently, Gaál et al. (2026) examined object-based contextual cueing using a paradigm modeled on Chun and Jiang (1999). They did not, however, observe a reliable cueing effect at the group level when spatial regularities were eliminated, reporting substantial individual variability instead. Taken together, these findings suggest that object-based contextual cueing may be more difficult to reproduce than initially assumed and that its emergence may depend critically on whether non-spatial information forms a stable and diagnostically informative context. One theoretically important but underexplored candidate for such a context is social information.

Although contextual cueing has been investigated extensively over the past two decades, research has rarely examined socially meaningful information. This absence is notable because social cues enjoy a privileged status in human perception. For example, faces signaling potential interaction reach awareness more readily (Fu et al., 2024), facing dyads are detected faster (Vestner et al., 2019) and attract more fixation (Skripkauskaite et al., 2023), and social interaction cues modulate emotion perception (Abramson et al., 2021; Gray et al., 2017). In memory as well, interacting individuals can become perceptually grouped or “chunked” (Ding et al., 2017; Paparella & Papeo, 2022). These findings collectively suggest that socially meaningful information is processed rapidly, prioritized automatically, and integrated into predictive mechanisms. Such properties may render social relational structure uniquely capable of providing stable and diagnostic predictive information, potentially counteracting the instability observed in object-based contextual cueing without spatial consistency.

Despite the privileged status of social information in human perception, it remains unclear whether socially meaningful cues can serve as contextual cues that guide attention in visual search. Although contextual cueing has been demonstrated across a broad range of stimulus domains, including natural scenes (e.g., Brockmole & Henderson, 2006; Brockmole et al., 2006), most prior work has relied on displays that lack inherent social meaning, such as letters, geometric shapes, or objects. As a result, it is still unknown whether the visual system can implicitly learn recurring social patterns and use them as contextual cues, particularly when spatial regularities are eliminated.

A recent study by Nagy et al. (2024) represents an initial attempt to introduce socially relevant stimuli into the contextual cueing paradigm by incorporating faces into the search displays. However, in that study, predictive context was defined by repeated spatial configurations, while the social properties of the stimuli themselves—such as facial identity or emotional expression—were not systematically varied. Thus, an important question remains open: can social information itself be implicitly learned and used to guide attention, independent of spatial context? Gaál et al. (2026) provide a further relevant test by using face stimuli with the aim of examining object-based contextual cueing in the absence of fixed spatial layouts. Although their study moved beyond abstract shapes to socially meaningful stimuli, they did not observe a reliable contextual cueing effect. Rather than resolving the broader debate on object-based contextual cueing, these findings highlight the need to more directly examine whether specific forms of social information can function as contextual cues when spatial regularities are eliminated.

To address this question, we conducted three experiments in which spatial layouts were fully randomized on every trial to prevent location-based learning. Instead, each experiment isolated a different form of social information that could, in principle, serve as a contextual cue. In the first experiment, the relevant structure was the stable association between a target identity and a set of distractor identities. In the second, the cue was the overall mood of the display, quantified as the ratio of angry to happy faces. In the third, we focused on relational structure—specifically, the pattern of who was facing whom within pairs of profile-view faces. Critically, these three cues vary in abstraction and perceptual processing demands. Identity associations rely on fine-grained recognition of individual faces; ensemble emotion depends on rapid extraction of global summary statistics (Chong & Treisman, 2003); and facing direction requires encoding relational configurations among elements. Examining these distinct forms of information allowed us to assess whether contextual learning generalizes across multiple levels of social meaning and whether some types are more readily acquired than others. Across all experiments, we compared performance under consistent versus variable social mappings and assessed whether learning occurred implicitly. Rather than merely extending the stimulus domain, the present study proposes that social relational structure may act as a predictive scaffold that stabilizes object-based contextual learning even in the absence of spatial consistency. By examining various levels of social meaning, we aim to identify the specific conditions under which non-spatial, object-based contextual cueing reliably emerges, thereby addressing the inconsistencies reported in recent replication attempts (e.g., Gaál et al., 2026; Makovski, 2016).

## Experiment 1

Experiment 1 examined whether stable associations between a target face and a set of distractor identities can support contextual learning when spatial regularities are eliminated. Unlike the geometric shapes used in Chun and Jiang’s (1999) paradigm, we employed front-facing facial stimuli with neutral expressions. In the Consistent Mapping condition, each target face was paired with the same set of distractor faces across the experiment. In the Variable Mapping condition, target–distractor pairings were randomized across blocks. On every trial, the spatial configurations of stimuli were randomized to eliminate predictive spatial structure. If participants can implicitly acquire the identity associations and use them to guide attention, visual search should become faster in Consistent Mapping trials than in Variable Mapping trials as the experiment progresses. Awareness tests were included to evaluate whether any learning was explicit or implicit.

## Method

### Participants

Twenty undergraduate students (1 man and 19 women; *M*_age_ = 21.9 years) with normal or corrected-to-normal vision participated. Participants were recruited through the university’s SONA online participant pool system and received course credit as compensation (1 credit per 30 min of participation). All provided written informed consent before the experiments began, and the study was approved by the Institutional Review Board of Yonsei University.

Instead of conducting a frequentist power analysis, the sample size was determined using a Bayesian optional stopping rule (Rouder, 2014). According to this rule, data collection began with a minimum of 10 participants, and the Bayes factor (BF_10_) for the Mapping effect was monitored at increments of five participants. Recruitment ceased once the BF_10_ exceeded approximately 10, indicating strong support for the alternative hypothesis, or when the maximum of 30 participants was reached. Thus, the final sample fell within the planned range of 10–30 participants.

### Apparatus and stimuli

The experiment was programmed in PsychoPy Version 3 (Peirce et al., 2019) and presented on a 24-in. LED monitor (1920 × 1080 resolution, 60 Hz) at a viewing distance of 60 cm. Participants’ heads were stabilized using a chin-and-forehead rest to ensure a consistent eye-to-screen distance. All stimuli were presented at a fixed size on a gray background.

A total of 96 front-facing facial photographs with neutral expressions were used: thirty-six images were obtained from the Radboud Faces Database (RaFD; Langner et al., 2010) and 60 from the Lifespan Database of Adult Facial Stimuli (Minear & Park, 2004), with prior permission. Sixteen female faces served as targets, and the remaining 80 male faces were used as distractors. Because perceived femininity of target could influence task performance, all target faces were pre-rated for perceived femininity on a 7-point Likert scale by an independent group of 10 participants (5 men, 5 women). Based on these ratings, target faces were counterbalanced across Mapping conditions to ensure similar femininity levels. Additional details about the rating procedure are provided in the Supplemental Material (Sect. 1). All images were cropped to the face region and subtended 3.0° × 4.0° of visual angle. 

On each trial, one target face and ten distractor faces (11 faces in total) were presented simultaneously. Stimuli were displayed within a centrally defined rectangular region (1397 × 974 pixels) subdivided into a 6 × 8 grid, yielding 48 possible stimulus locations corresponding to the centers of the grid cells. For each trial, 11 locations were randomly selected from these grids, and the target and distractors were assigned to distinct positions. The centers of immediately neighboring grids were separated by approximately 175 pixels horizontally and 162 pixels vertically, preventing spatial overlap between face stimuli. To introduce positional variability while preserving this minimum spacing, spatial jitter was applied within the non-overlapping range (horizontal: 0–24 pixels in 3-pixel increments; vertical: 0–3 pixels). Given the image size (117 × 155 pixels), this resulted in a minimum edge-to-edge spacing of approximately 34 pixels horizontally and 4 pixels vertically between face images. The target location was not constrained to any specific grid location, and the spatial positions of all stimuli, including the target and distractors, were randomly determined on each trial without additional spatial constraints.

### Procedure

Each trial required participants to search for a female face (target) among 10 male faces (distractors) as quickly and accurately as possible. Each display included 10 distractors drawn from five distinct male identities, with identities repeated up to three times per display. Eight epochs were administered, each consisting of five blocks of 16 trials (80 trials per epoch). Within each block, trials were evenly divided between the Consistent and Variable Mapping conditions (eight trials per condition). The 16 target identities were evenly divided between the two Mapping conditions, and the 80 distractor identities were randomly split into two non-overlapping pools of 40. In the Consistent Mapping condition, each target was paired with a fixed set of five distractor identities, with these pairings assigned randomly for each participant. In the Variable Mapping condition, the five distractor identities paired with each target changed on every block, drawn without replacement from the corresponding 40-identity pool. Randomizing the distractor identities on each block prevented participants from forming stable identity-based associations. Across both conditions, each target and each distractor identity appeared only once per block within its assigned condition. This ensured that identity exposure was fully equated within each condition, preventing familiarity differences from confounding performance and isolating the effect of consistent versus inconsistent target–distractor relations. No separate practice trials were conducted prior to the main experiment.

**Fig. 1:**
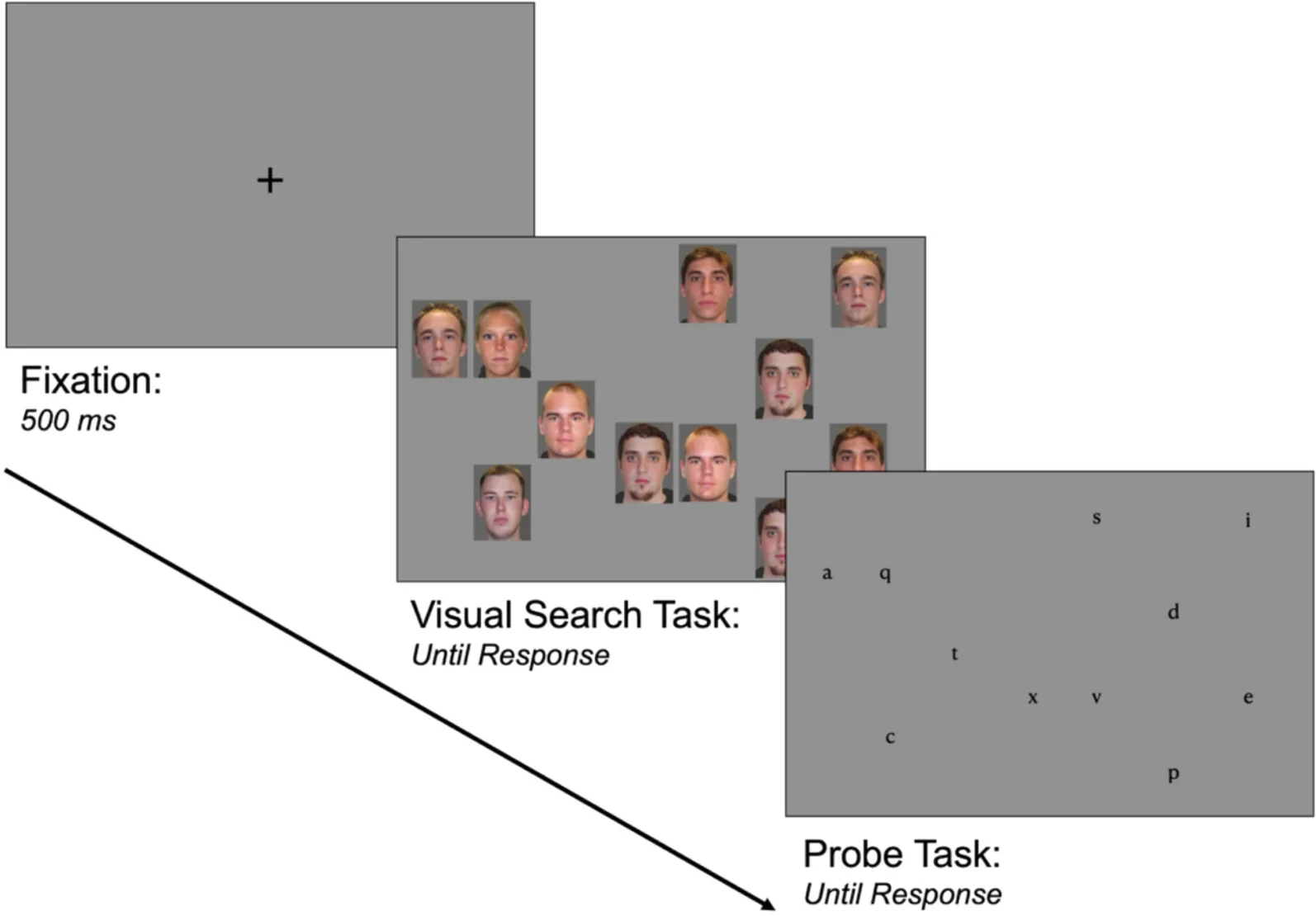
The schematic trial sequence of Experiment 1. Trial sequence. Each trial began with a 500-ms fixation cross, followed by a visual search display containing one female target and 10 male distractors. Trials were assigned to either the Consistent or Variable Mapping condition. Participants responded by pressing the spacebar as soon as they located the target, and the display remained visible until response. Immediately afterward, a probe display appeared with letters in all previous face locations, and participants typed the letter that had appeared at the target’s location. Auditory feedback was provided for incorrect responses

Figure 1 illustrates the trial sequence. Each block began with a spacebar press, followed by a 500-ms fixation cross. A visual search display containing 11 faces then appeared and remained until response. Participants responded by pressing the spacebar as soon as they located the target. Immediately after the response, the faces disappeared and were replaced by letter probes at the previous face locations. Letters were randomly selected from the alphabet (excluding “l”), and participants typed the letter that had appeared at the target’s location. The target letter was not repeated on consecutive trials. This probe task verified target localization accuracy, with auditory feedback provided for incorrect responses.

After the visual search task, participants performed two awareness tests. In the recognition test, they were informed that some targets might have been consistently paired with specific distractors during the experiment and were asked whether they had noticed any such regularities. Responses were recorded as a binary choice (yes or no). No face images were presented during the recognition test. Following the recognition test, participants completed a forced-choice generation task designed to assess explicit knowledge of the target–distractor pairings. Only face identities that had appeared in the Consistent Mapping condition during the visual search task were used. The task consisted of eight trials, corresponding to the eight target identities in the Consistent Mapping condition. On each trial, five male distractor faces were presented simultaneously in a horizontal row, and eight female target faces from the Consistent Mapping condition were presented simultaneously in a separate horizontal row, each accompanied by a numerical label. All face images were presented at the same size as in the visual search task. Participants were asked to select the female face they believed had been paired with the displayed distractors by pressing the corresponding number key. The display remained on the screen until the participant responded. The forced-choice trials were presented in a randomized order, and no feedback was provided regarding response accuracy in either awareness test.

### Data analysis

Response times (RTs) from the visual search task were analyzed only for trials with correct probe responses. Trials with RTs shorter than 200 ms or beyond + 2.5 SDs of a participant’s mean were excluded. RTs were then averaged within each condition for each participant. To test the hypothesized contextual cueing effects, we conducted Bayesian repeated-measures ANOVAs with Mapping (Consistent vs. Variable) and Epoch (1–8) as a within-subject factor. Additionally, to examine changes across broader stages of the experiment, Epochs were collapsed into a Phase factor with two levels: Early (Epochs 1–4) and Late (Epochs 5–8). Bayesian analyses were performed using JASP (JASP Team, 2024) and RStudio (RStudio Team, 2020). Bayes factors (BF_10_ or BF_incl_) quantified evidence for the alternative hypothesis relative to the null, with interpretive thresholds following Jeffreys (1961) and Lee and Wagenmakers (2013).

### Transparency and openness

We report how we determined our sample size, all data exclusion procedures, all manipulations, and all measures in accordance with the Journal Article Reporting Standards (Appelbaum et al., 2018). All data and analysis code for all experiments are publicly available on the Open Science Framework (https://osf.io/etz9w/?view_only=19963df218d041be8e3e781df99226b1). The stimulus materials cannot be shared publicly because they were developed by other researchers and used here with permission from the copyright holders. This study was not preregistered.

## Results and discussion

**Fig. 2:**
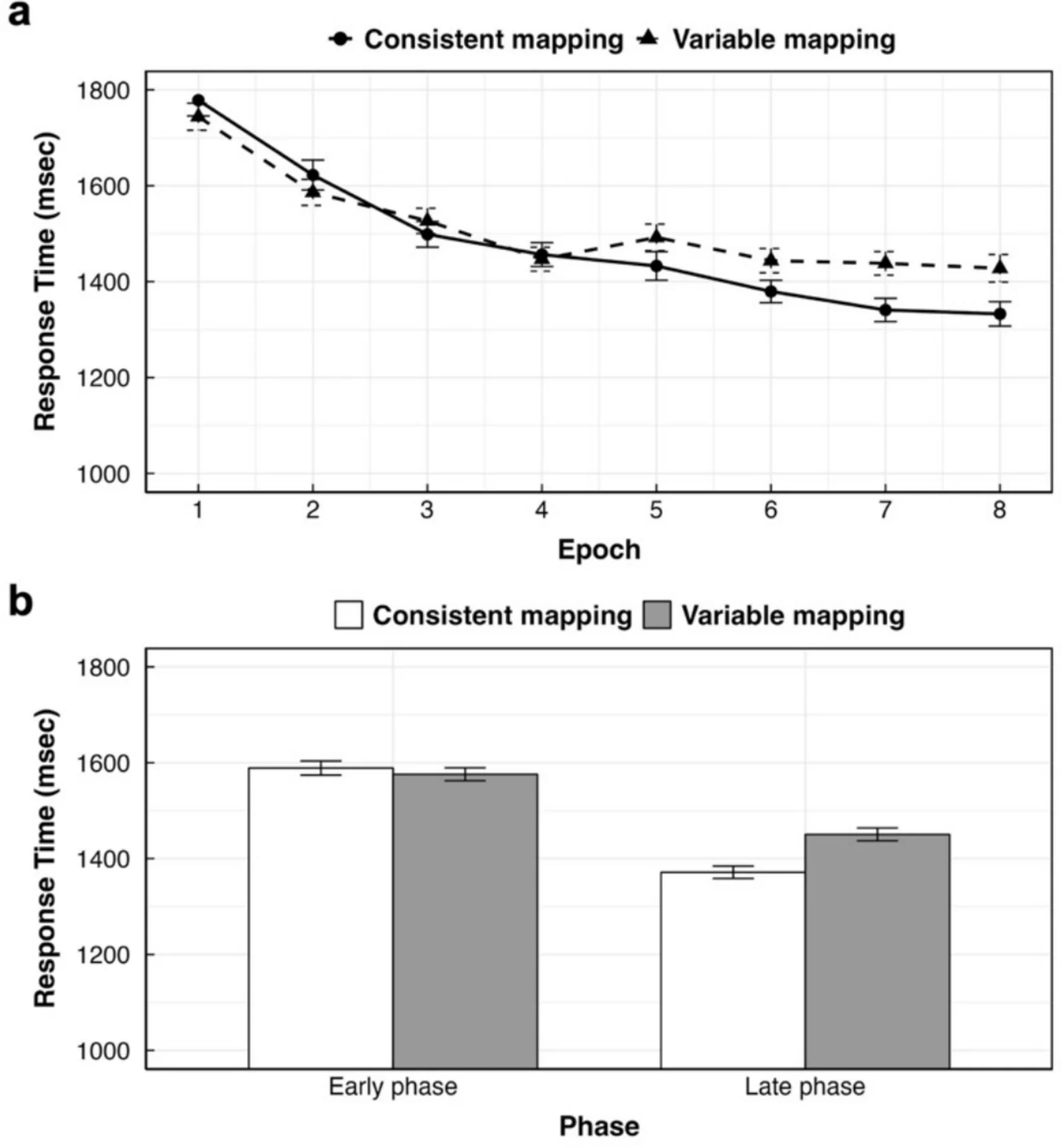
Results of experiment 1. **a** Mean RTs across eight Epochs for the Consistent and Variable Mapping conditions. **b** Mean RTs collapsed into Early (Epochs 1–4) and Late (Epochs 5–8) Phases. Error bars represent ± 1 standard errors of the mean

Overall accuracy was high (98.64%), with no participants excluded. Trials with RTs < 200 ms or beyond 2.5 SDs of a participant’s mean were removed, eliminating only 0.11% of the data. As shown in Fig. 2a, the model including only the main effect of Epoch best predicted the RT data (BF_10_ = 1.0 × 10^19^), providing decisive evidence relative to the null model. In contrast, the main effect of the Mapping (BF_incl_ = 0.50) and the Mapping × Epoch interaction (BF_incl_ = 0.89) yielded anecdotal evidence for the null hypothesis, indicating a lack of support for these effects.

To examine differences across time, Epochs were collapsed into an Early Phase (Epochs 1–4) and a Late Phase (Epochs 5–8) and analyzed with a 2 × 2 Bayesian repeated-measures ANOVA. This phase-based analysis was exploratory in nature and was not specified a priori; it was intended to provide a broad comparison between earlier and later stages of the task. The best-fitting model included both main effects and their interaction (BF_10_ = 1.25 × 10^7^). The main effect of Phase provided extremely strong evidence (BF_incl_ = 5.59 × 10^6^), the main effect of Mapping showed substantial evidence (BF_incl_ = 17.27), and their interaction provided very strong evidence (BF_incl_ = 62.27). As shown in Fig. 2b, RTs did not differ between Mapping conditions in the Early Phase (*M*_cons_ = 1.59 s, *SD*_cons_ = 0.19 s; *M*_var_ = 1.58 s, *SD*_var_ = 0.17 s). In the Late Phase, however, RTs were lower in the Consistent Mapping condition (*M*_cons_ = 1.38 s, *SD*_cons_ = 0.20 s) than in the Variable Mapping condition (*M*_var_ = 1.45 s, *SD*_var_ = 0.17 s). In spatial contextual cueing paradigms, learning often emerges early, with reliable effects observed by the second epoch (e.g., Chun & Jiang, 1998). In contrast, in the present experiment, the cueing effect became evident primarily in the later phase of the task, suggesting that learning based on non-spatial contextual information may unfold more gradually. Taken together, these results indicate that participants gradually learned and used the stable target–distractor associations, consistent with implicit learning of target–distractor face pairings as contextual cues.

## Awareness tests

In the recognition test, no participant reported noticing consistent target–distractor pairings. In the forced-choice generation test, the mean accuracy across all participants was 11.88%, which did not exceed the chance level of 12.5%. Five participants performed above the chance level (*M* = 27.5%), whereas the remaining 15 participants scored below chance (*M* = 6.67%). When the five above-chance participants were excluded, the Phase-based analysis yielded a similar pattern: anecdotal evidence for a main effect of Mapping (BF_incl_ = 2.33), and substantial evidence for its interaction with Phase (BF_incl_ = 7.78). These findings suggest that the contextual cueing effect in the Consistent Mapping condition is more consistent with implicit learning than with explicit knowledge.

## Experiment 2

Experiment 1 demonstrated that participants could learn identity-based target–distractor associations that facilitated search. Building on this, Experiment 2 investigated whether the overall mood of the display—an ensemble level social cue—could serve as a contextual cue. This cue was based on ensemble coding (Chong & Treisman, 2003), which allows observers to rapidly extract statistical information from groups of faces. Prior work has shown that ensemble coding supports perception of identity (de Fockert & Wolfenstein, 2009), gender (Haberman & Whitney, 2007), and emotion (Haberman & Whitney, 2009). Recent evidence indicates that observers can accurately perceive the ratio of angry to happy faces (Son et al., 2023), suggesting that such emotional ratios are both perceptually accessible and potentially learnable.

To test whether the overall emotional tone could serve as a contextual cue, both target and distractor faces displayed either angry or happy expressions, and each trial was defined by an overall mood ratio ranging from 1:14 to 14:1 (angry-to-happy faces). Of the 14 distinct ratios, half were consistently paired with a specific target (Consistent Mapping), and the other half varied randomly across trials (Variable Mapping). As in Experiment 1, spatial arrangements were fully randomized to eliminate location-based learning. If global information can be implicitly associated with target identities, then stable angry-to-happy ratios should speed visual search in Consistent Mapping trials relative to Variable Mapping trials.

## Method

### Participants

Sixteen participants (3 men and 13 women; *M*_age_ = 23.2 years) with normal or corrected-to-normal vision took part in Experiment 2. None had participated in Experiment 1. Participants were recruited using the same procedure as in Experiment 1 and received course credit for their participation. As in Experiment 1, sample size determination followed a Bayesian optional stopping rule. Data collection began with a minimum sample size, and the Bayes factor (BF_10_) for the Mapping condition effect was monitored in increments of five participants. Recruitment was planned to terminate once the BF_10_ exceeded approximately 10 or when a predetermined maximum sample size was reached. The final sample size slightly exceeded the planned stopping point because participants had already been scheduled on a daily basis.

### Apparatus and stimuli

Apparatus and display settings were identical to those used in Experiment 1. Stimuli consisted of 56 front-facing facial photographs from the RaFD (Langner et al., 2010), derived from 14 female and 14 male identities. Each identity was photographed with both happy and angry expressions, yielding 28 female images (used as targets) and 28 male images (used as distractors). To minimize variability between target and distractor faces, all stimuli were drawn from the same database and were restricted to Caucasian identities. Consequently, all target and distractor faces used in Experiment 2 were drawn from the same set of identities used in Experiment 1. All images were presented at 3.0° × 4.0° of visual angle on a gray background, as in Experiment 1. The spatial arrangement of stimuli, including the grid-based layout, spacing constraints, and positional jitter, was identical to that used in Experiment 1.

### Procedure

**Fig. 3:**
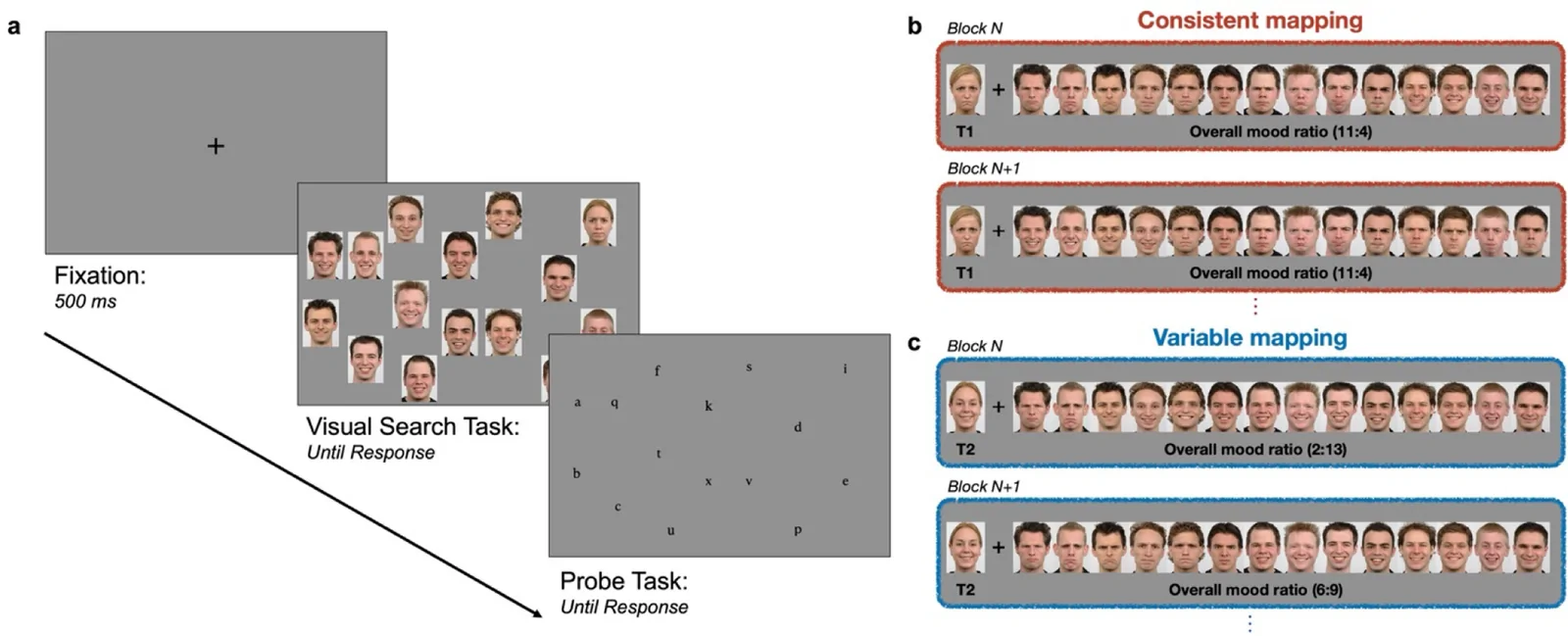
Schematic trial sequence and example displays for the Mapping conditions in Experiment 2. **a** Trial sequence. Each trial began with a 500-ms fixation cross, followed by a search display containing 15 faces (1 female target, 14 male distractors). Participants responded by pressing the spacebar upon locating the target, after which a probe display appeared at the target location for letter identification. Auditory feedback was provided for incorrect responses. **b** Example configurations for the Consistent Mapping condition. A target was always paired with a fixed overall mood ratio (e.g., 11 angry to 4 happy faces) across all blocks, whereas the assignment of emotional expressions to individual male faces was randomized across blocks. For example, certain male faces may have appeared angry in one block, while different male faces appeared angry in the next block. **c** Example configurations for the Variable Mapping condition. The target–mood ratio pairing changed across blocks, such that a target appeared with one ratio in a given block (e.g., 2 angry to 13 happy faces) and a different ratio in the subsequent block (e.g., 6 angry to 9 happy faces)

The procedure was identical to that of Experiment 1 except as described below. The experiment consisted of eight epochs, each containing five blocks of 14 trials. Each display presented 15 faces: one female target and 14 male distractors (Fig. 3a). Trials were evenly divided between the Consistent and Variable Mapping conditions. In the Consistent Mapping condition, each target face was paired with a specific angry-to-happy ratio that remained constant across the experiment (Fig. 3b). For example, Target A would always appear in displays containing 11 angry and 4 happy faces. Although the target–ratio pairing remained fixed, the assignment of emotional expressions to individual identities was randomized at the beginning of each block, such that which specific faces appeared angry or happy varied from block to block. In the Variable Mapping condition, target–ratio pairings varied randomly across blocks (Fig. 3c). For example, Target B would appear with a 2:13 angry-to-happy ratio in one block and a 6:9 ratio in the next block, preventing the formation of a stable association between the target and any specific ratio. The 14 female target identities were assigned to the Consistent and Variable Mapping conditions using the same counterbalancing procedure as in Experiment 1, based on the pre-rating results reported in the Supplemental Material (Sect. 1). Each target identity appeared once per block in its assigned mapping condition, ensuring that target frequency was matched across conditions.

The mood ratios, defined as the number of angry versus happy faces in the display (including the target), ranged from 1:14 to 14:1, yielding 14 possible ratios. These ratios were divided into two sets of seven. Participants were randomly assigned to one of two counterbalancing groups. For Group A, the Consistent Mapping condition included the seven ratios with an odd number of angry faces (1:14, 3:12, 5:10, 7:8, 9:6, 11:4, 13:2), whereas the remaining ratios were assigned to the Variable Mapping condition (2:13, 4:11, 6:9, 8:7, 10:5, 12:3, 14:1). For Group B, this assignment was reversed. In the extreme ratios (1:14 and 14:1), the emotional singleton (one angry or one happy face) was always assigned to the target to ensure balanced target expressions across participants.

The same set of 14 distractor identities was used in both Mapping conditions, and only their emotional expressions varied. Importantly, emotional expressions were randomly assigned to individual face identities at the beginning of each block, even in the Consistent Mapping condition, such that which specific faces appeared angry or happy varied from block to block. Thus, no specific identity was consistently associated with a particular expression across repetitions. This design prevented identity-based associations, ensuring that any performance facilitation would primarily reflect the ratio rather than identity-based cues. Spatial arrangements were randomized on every trial, with positional jitter identical to that used in Experiment 1. No separate practice trials were conducted prior to the main experiment.

After completing the visual search task, participants completed the same two awareness tests as in Experiment 1. In the recognition test, participants indicated whether they had noticed any regularities during the experiment, with responses recorded as a binary choice (yes or no). Immediately thereafter, participants performed a forced-choice generation task designed to assess explicit knowledge of the target–ratio associations. This task consisted of 14 trials, corresponding to all mood ratios used in the experiment and thus included both Consistent and Variable Mapping trials. The target–mood ratio pairings tested in this task were drawn from a block in the middle of the visual search task, such that the Variable Mapping trials reflected the target–ratio assignments participants had encountered in that block. On each trial, participants were shown a set of 14 male distractor faces presented with a specific overall angry-to-happy ratio. As in the main visual search task, emotional expressions were randomly assigned to individual distractor identities on each trial. Participants were shown all 14 female target faces simultaneously and were asked to select the target face they believed had been presented together with the displayed distractors by pressing the corresponding number key. All target faces were presented with the same emotional expression within a trial, such that the emotional expression of the target and the distractors together instantiated the overall mood ratio associated with that trial. The trials were presented in a randomized order. No feedback was provided regarding response accuracy.

### Data analysis

RT data were preprocessed and analyzed with Bayesian repeated-measures ANOVAs using the same criteria as in Experiment 1.

## Results and discussion

**Fig. 4:**
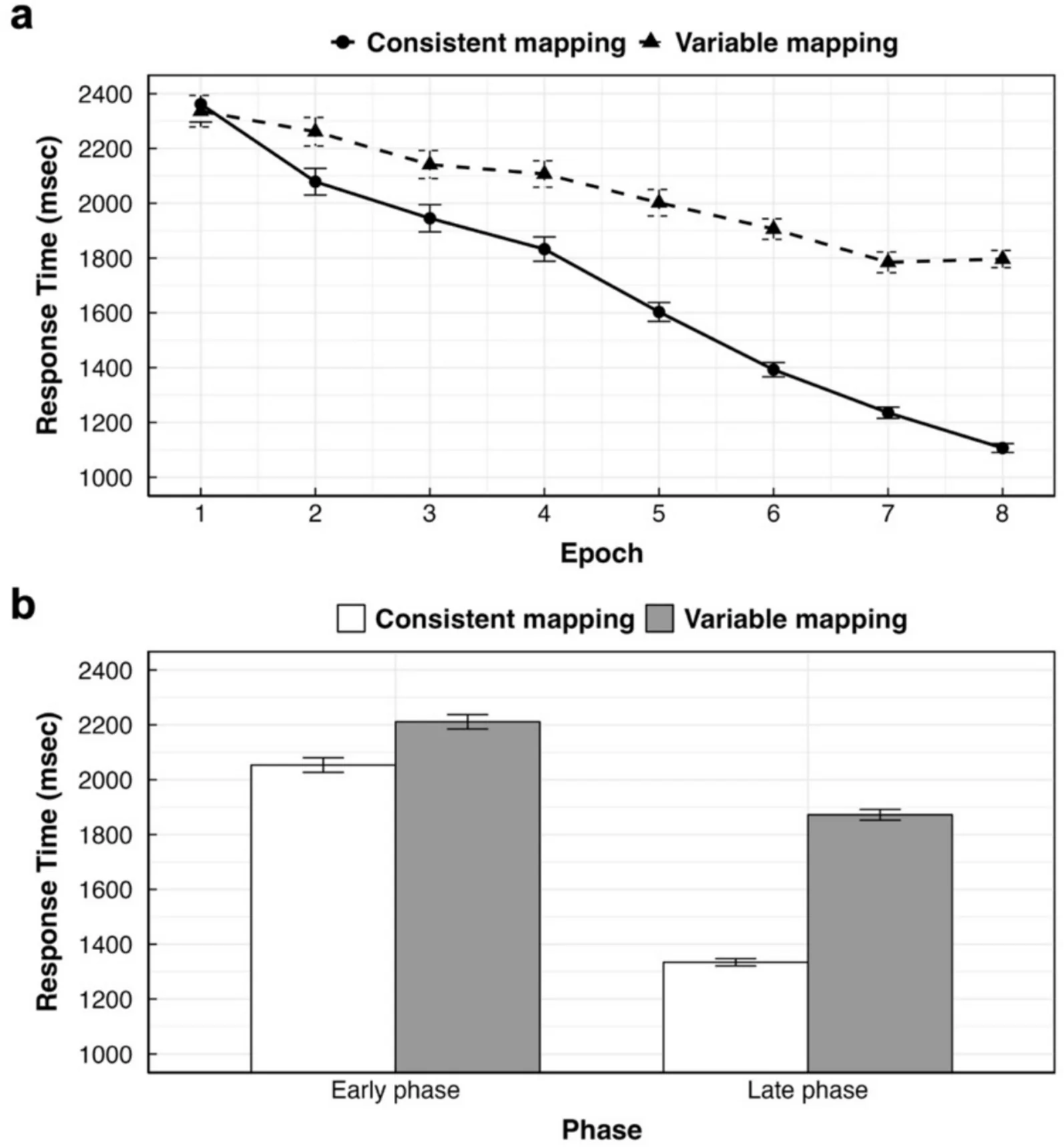
Results of experiment 2. **a** RTs across eight Epochs for the Consistent and Variable Mapping conditions. **b** RTs collapsed into Early (Epochs 1–4) and Late (Epochs 5–8) Phases for each Mapping condition. Error bars represent ± 1 SE

All 16 participants were included in the analysis (overall accuracy = 98.65%). Applying the same RT exclusion criteria as in Experiment 1 resulted in the removal of 0.20% of the data. As shown in Fig. 4a, the Bayesian repeated-measures ANOVA across the eight Epochs revealed that the model including both main effects and their interaction provided the best account of the data (BF_10_ = 1.93 × 10^42^). The interaction term showed extremely strong evidence (BF_incl_ = 7.23 × 10^9^), indicating that performance changed over time depending on Mapping condition.

When Epochs were grouped into Early (1–4) and Late (5–8) Phases (Fig. 4b), the full model again provided the best fit (BF_10_ = 9.75 × 10^13^), with extremely strong evidence for the interaction (BF_incl_ = 1.06 × 10^5^), as well as for the main effects of Mapping (BF_incl_ = 2.26 × 10^8^) and Phase (BF_incl_ = 2.74 × 10^10^). In the Early Phase, RTs were similar across conditions (*M*_cons_ = 2.06 s, *SD*_cons_ = 0.28 s; *M*_var_ = 2.21 s, *SD*_var_ = 0.21 s). In the Late Phase, RTs were faster in the Consistent Mapping condition (*M*_cons_ = 1.33 s, *SD*_cons_ = 0.21 s) than in the Variable Mapping condition (*M*_var_ = 1.87 s, *SD*_var_ = 0.21 s). In contrast to Experiment 1, this pattern indicates that learning emerged relatively early in the task, with participants rapidly acquiring and using the consistent mood ratio as a contextual cue to guide attention toward the target. To rule out the possibility that the facilitation was driven by particular mood ratios (e.g., extreme ratios such as 1:14 or 14:1), we examined reaction times across all 14 ratios. Neither the main effect of counterbalancing group nor that of the ratios was observed (see Supplemental Material Sect. 2). Thus, the effect cannot be explained by certain ratios being inherently easier but instead reflects learning of the stable target–overall mood ratio pairing.

## Awareness tests

In the recognition test, 9 of the 16 participants reported noticing a consistent target–mood ratio association. In the forced-choice generation task, accuracy was computed only for trials corresponding to the Consistent Mapping condition, for which a unique target-mood ratio paring was defined (chance level = 7.14%). Across all participants, mean accuracy on these trials was 4.46%. Five participants performed above chance (*M* = 14.3%), whereas the remaining 11 participants scored below chance.

When the five above-chance participants were excluded, results were unchanged: in the eight-Epoch analysis, the full model remained dominant (BF_10_ = 1.62 × 10^28^), with strong evidence for the interaction term (BF_incl_ = 7.55 × 10^9^). The Phase-based analysis also showed strong evidence for the interaction (BF_incl_ = 4.98 × 10^4^) and for the main effect of the Mapping (BF_incl_ = 8.24 × 10^5^). These findings suggest that the learning effects primarily reflected implicit learning rather than explicit awareness of the target–mood ratio associations.

## Experiment 3

Experiment 3 examined whether a relational social cue—the pattern of who is facing whom—can support contextual learning when spatial regularities are eliminated. Prior research has shown that facing dyads are processed efficiently and tend to be grouped perceptually, reflecting their social relevance (Vestner et al., 2019). Unlike Experiments 1 and 2, which relied on identity-based or ensemble-based properties of the display, this experiment tested whether participants could implicitly learn relational configurations among pairs of faces and use this information to guide visual search. Because relational cues require integrating directional information across multiple pairs, they may impose greater cognitive demands than ensemble emotion but could nevertheless support contextual cueing if observers extract these structures automatically. If so, performance should become faster in Consistent Mapping trials compared to Variable Mapping trials over time.

## Method

### Participants

Twenty participants (5 men and 15 women; *M*_age_ = 22.8 years) with normal or corrected-to-normal vision took part in Experiment 3. None had participated in Experiments 1 or 2. Participants were recruited using the same procedure and stopping rule as in the previous experiments and received course credit for their participation.

### Apparatus and stimuli

Apparatus was identical to that of Experiment 1. Stimuli consisted of 28 profile-view facial photographs from the RaFD (Langner et al., 2010): 14 female faces served as targets and 14 male faces as distractors, all with neutral expressions. The identities of both target and distractor faces were drawn from those used in Experiment 2; only facial expression and viewing angle differed. The same 14 distractor identities were used on every trial in both mapping conditions, and each target appeared once per block. Stimuli were displayed within the same centrally defined rectangular region used in Experiments 1 and 2. In Experiment 3, this region was subdivided into a 5 × 8 grid, resulting in 40 grid locations defined by the centers of the grid cells. Grid cell size was increased relative to the previous experiments, yielding fewer but larger cells while preserving the overall display area. Within each grid cell, two stimulus positions were defined to allow two faces to be presented in close horizontal proximity, reflecting the requirement in Experiment 3 that faces be grouped into pairs. The two positions within a cell were separated by approximately 117 pixels horizontally, forming a local pair. The centers of immediately neighboring grid cells were separated by approximately 232 pixels horizontally and 195 pixels vertically, ensuring sufficient spacing between different face groups and preventing spatial overlap between stimuli. As in the previous experiments, spatial jitter was applied to the selected positions to introduce positional variability without violating these spacing constraints. Stimulus size, background color, and other display settings were identical to those used in the previous experiments.

### Procedure

**Fig. 5:**
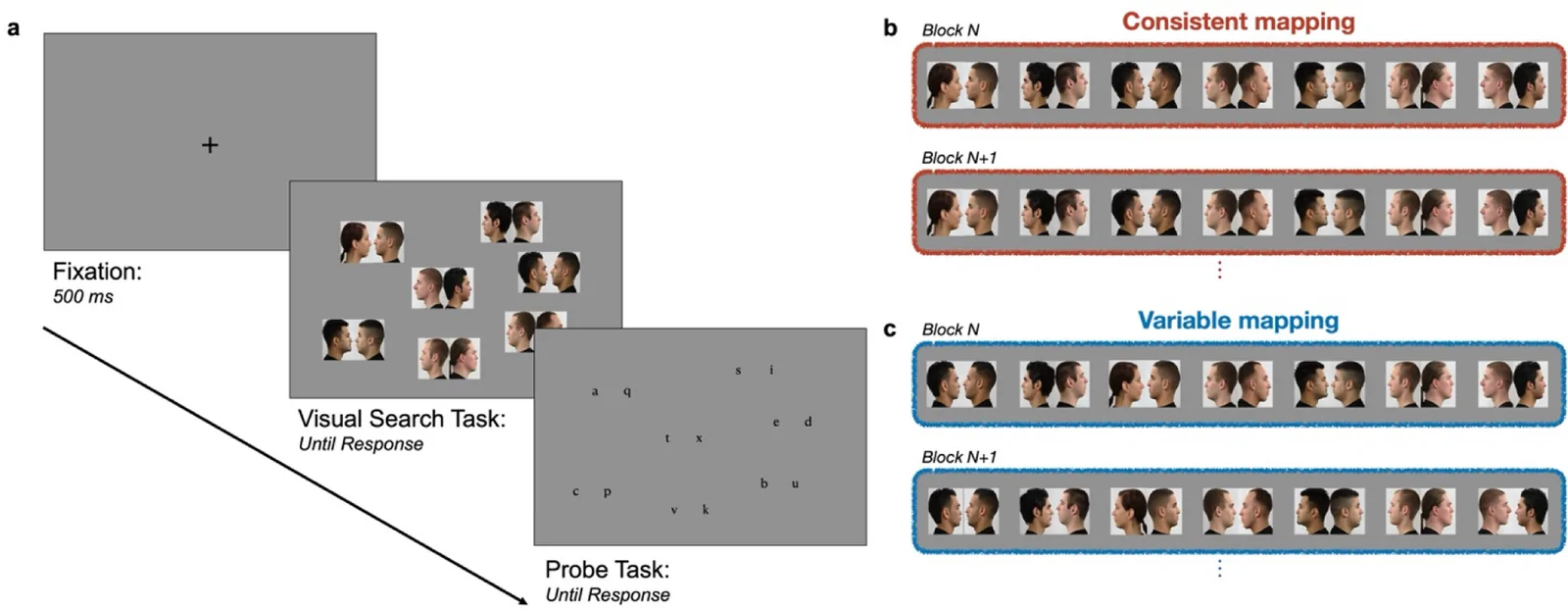
Schematic trial sequence and example displays for the mapping conditions in experiment 3. **a** Trial sequence. Each trial began with a 500-ms fixation, followed by a search display containing 14 faces (7 pairs; 1 female target and 13 male distractors), and then a probe display. Auditory feedback was provided for incorrect responses. **b** Example configurations for the Consistent Mapping condition. The same seven face pairs and their facing directions remained constant across all blocks (i.e., which pairs faced toward or away from each other). **c** Example configurations for the Variable Mapping condition. The same seven face pairs were maintained, and the overall ratio of facing to non-facing pairs (3:4 or 4:3) was held constant within each block, but the assignment of facing directions within pairs varied randomly across blocks, with only a subset of pairs changing between successive configurations

The procedure was identical to that of Experiments 1 and 2 except as noted below (see Fig. 5a for the trial sequence). The experiment consisted of eight epochs, each containing five blocks of 14 trials. Each display contained 14 faces—1 target and 13 distractors—arranged into seven pairs. In this experiment, the contextual cue was defined by the relational facing directions of pairs of faces rather than by their spatial positions. Although the spatial location of each face varied randomly across trials, the pattern of which pairs faced toward or away from each other remained stable in the Consistent Mapping condition. Within each pair, the two faces either faced each other (facing) or faced away from each other (non-facing). To balance displays and avoid simple repetition of global configurations, each display contained either three facing and four non-facing pairs or four facing and three non-facing pairs, such that the overall ratio of facing to non-facing pairs (3:4 or 4:3) was preserved across conditions.

Trials were evenly divided between the Consistent and Variable Mapping conditions, which differed in how the facing-direction configuration of the seven pairs was associated with the target. In the Consistent Mapping condition, each target was paired with a fixed facing-direction configuration that remained constant across all blocks of the experiment. For example, if Target A appeared facing Distractor A while another pair (e.g., Distractors B and C) appeared non-facing in one block, this specific pattern of facing and non-facing pairs was preserved throughout the experiment (Fig. 5b). In the Variable Mapping condition, the pairing between the target and the facing-direction configuration changed from block to block. For example, a configuration in which Target B faced Distractor D and Distractors E and F were non-facing in one block could be replaced in the next block by a configuration in which Target B and Distractor D were non-facing while Distractors E and F were facing (Fig. 5c). Across both conditions, the pairing of face identities remained fixed (e.g., Target A–Distractor A, Target B–Distractor D, Distractors B–C, Distractors E–F), and only the facing directions within these fixed pairs varied according to the Mapping condition.

To prevent participants in the Variable Mapping condition from learning unintended regularities (such as all pairs reversing direction simultaneously), an additional control was applied. On each Variable Mapping trial, the facing direction of 3–5 of the seven pairs was flipped relative to the previous configuration, while preserving the overall ratio of facing to non-facing pairs (3:4 or 4:3). Spatial positions were randomized on each trial, and positional jitter was applied in the same manner as in Experiment 1. The experiment began without any separate practice trials.

After the visual search task, participants completed the same two awareness tests as in Experiments 1 and 2. The recognition test assessed self-reported awareness of regularities using a binary (yes/no) response. Immediately following the recognition test, participants performed a forced-choice generation task designed to assess explicit knowledge of the target-facing configuration associations. This task included trials corresponding to both the Consistent and Variable Mapping conditions. On each trial, participants were shown 13 distractor faces. Of these, the distractor that had been paired with the target during the visual search task was presented alone, whereas the remaining distractors were presented as six face pairs, preserving the original pairing structure. In trials corresponding to the Consistent Mapping condition, the facing directions assigned to each distractor pair during the visual search task were preserved. Thus, pairs that had consistently appeared facing each other or facing away from each other during the search task were presented with the same facing configuration in the awareness task. In trials corresponding to the Variable Mapping condition, distractor-facing configurations were taken from a block in the middle of the visual search task. At the same time, all 14 target faces were presented simultaneously. Participants were instructed to select the target face they believed had been presented together with the displayed distractors by pressing the corresponding number key. The facing direction of each target face matched the facing direction that had been assigned to its paired distractor in the visual search task for the corresponding trial type. Trial order in the forced-choice task was randomized, and no feedback was given.

### Data analysis

RT data were preprocessed and analyzed with Bayesian repeated-measures ANOVAs using the same criteria and procedures as in Experiment 1.

## Results and discussion

**Fig. 6:**
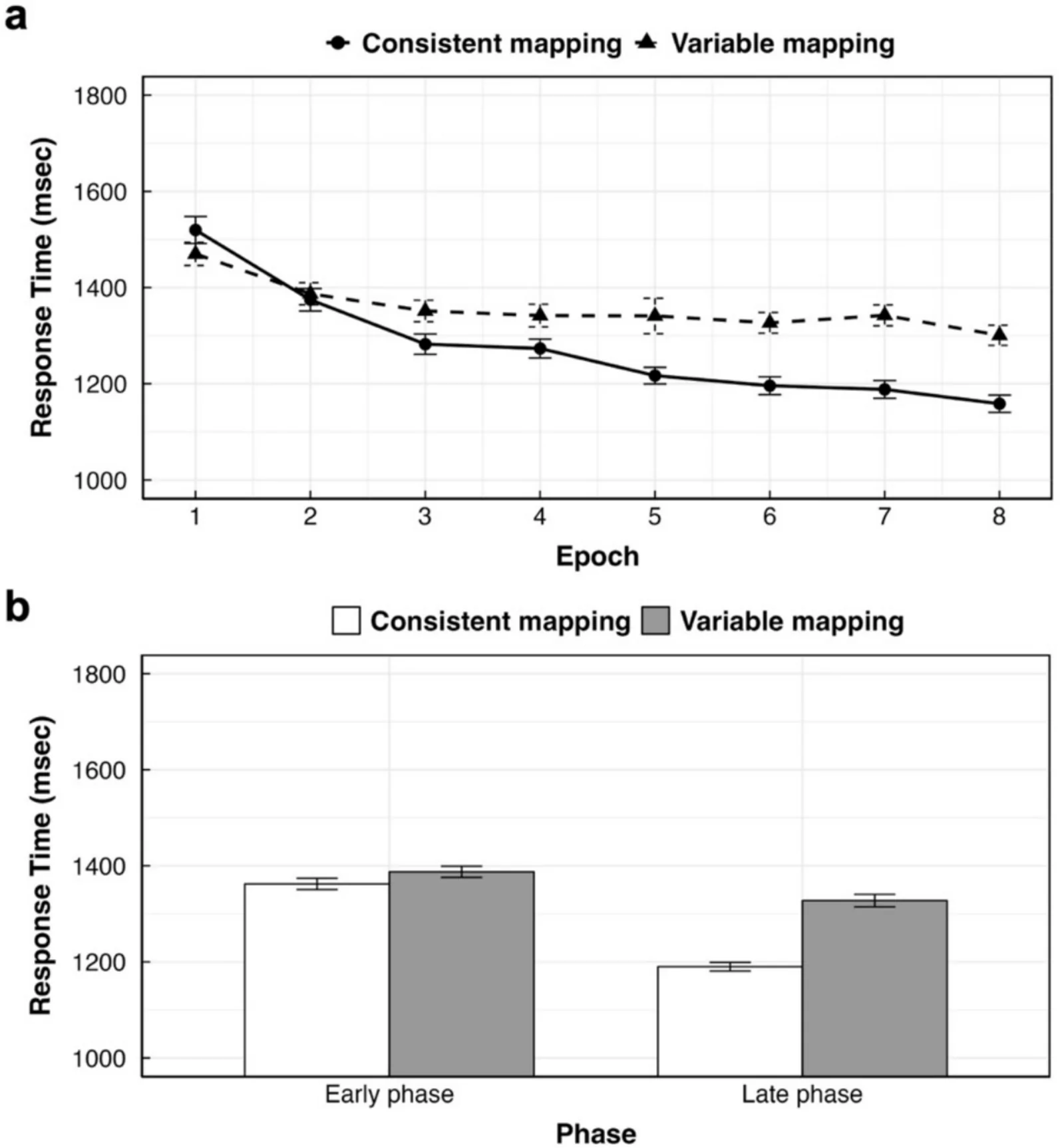
Results of experiment 3. **a** RTs across eight Epochs for the Consistent and Variable Mapping conditions. **b** RTs collapsed into Early (Epochs 1–4) and Late (Epochs 5–8) Phases for each Mapping condition. Error bars represent ± 1 SE

Overall accuracy was 98.61%, and no participants were excluded. Applying the same preprocessing criteria as in Experiments 1 and 2 led to the removal of 0.16% of the data. In the 8-Epoch analysis (Fig. 6a), the model including both main effects and their interaction provided the best account of the data (BF_10_ = 3.90 × 10^18^), with extremely strong evidence for the main effect of Mapping condition (BF_incl_ = 7.86 × 10^3^) and for the interaction (BF_incl_ = 7.57 × 10^2^). In the Phase-based analysis (Fig. 6b), the full model again emerged as the best fit (BF_10_ = 5.61 × 10^7^), with extreme evidence for the interaction between Phase and Mapping condition (BF_incl_ = 1.69 × 10^3^) and strong evidence for the main effect of Mapping condition (BF_incl_ = 2.28 × 10^4^). RTs were similar across conditions in the Early Phase (*M*_cons_ = 1.36 s, *SD*_cons_ = 0.12 s; *M*_var_ = 1.39 s, *SD*_var_ = 0.11 s), but in the Late Phase, RTs were shorter in the Consistent condition (*M*_cons_ = 1.19 s, *SD*_cons_ = 0.10 s) than in the Variable Mapping condition (*M*_var_ = 1.33 s, *SD*_var_ = 0.13 s). Similar to the results of Experiment 1, the emergence of this difference primarily in the later phase suggests that learning of the stable target–display associations developed gradually over time. These results indicate that participants learned the stable associations and used them to reach the decision that an item was the target more quickly.

## Awareness tests

In the recognition test, 9 participants reported noticing the regularities, and 10 participants performed above chance in the forced-choice generation test (overall mean accuracy = 8.57%, mean accuracy of the above-chance group = 17.14%, chance level = 7.14%). To assess whether the observed contextual cueing effect depended on explicit knowledge, we repeated the analyses after excluding these 10 participants. In the 8-Epoch analysis, the model including both main effects and their interaction remained strongly preferred (BF_10_ = 1.28 × 10^6^) with evidence for the main effect of Mapping condition (BF_incl_ = 3.81) and for the interaction (BF_incl_ = 8.85). Similarly, in the Phase-based analysis, the full model was again preferred (BF_10_ = 92.12), with evidence for both the main effect of the Mapping condition (BF_incl_ = 8.86) and the interaction (BF_incl_ = 20.89). Notably, however, the magnitude of the contextual cueing effect was reduced after excluding participants with above-chance performance. Taken together, these results suggest that although contextual cueing in Experiment 3 is not fully eliminated when participants with explicit knowledge are excluded, explicit awareness may have contributed to the observed effect to some extent and therefore cannot be entirely ruled out.

## General discussion

Across three experiments, the present study shows that socially meaningful information can function as contextual cues in visual search when spatial regularities are eliminated. Each experiment manipulated a different type of social cue—identity associations (Experiment 1), ensemble emotion (Experiment 2), and relational facing-direction pattern (Experiment 3)—while ensuring that spatial arrangements provided no predictive value. In all three experiments, participants showed faster search performance in Consistent Mapping conditions than in Variable Mapping conditions, indicating that they implicitly acquired and used stable social regularities to guide attention. These results suggest that contextual cueing mechanisms can incorporate socially meaningful information when it forms a stable and diagnostically informative relational structure, even in the absence of spatial regularities. Notably, the cues in Experiments 2 and 3 were task-irrelevant, yet still facilitated learning, consistent with recent evidence that contextual cueing can occur without task relevance (Tavera & Haider, 2025).

A key contribution of this work is the demonstration that different forms of social information—ranging from identity pairings to global emotional tone and relational orientation—can each support contextual learning. Prior contextual cueing research has focused predominantly on spatial regularities or object-level visual features (Chun & Jiang, 1998, 1999), with relatively few studies exploring higher-level semantic or social cues. The present findings show that contextual learning mechanisms are flexible and can incorporate abstract social structures that imply potential interaction. This suggests that the cognitive system may be tuned to recognize regularities in social environments and use them to facilitate behavior even when they are not explicitly attended.

Although all three types of social cues supported learning, their effectiveness differed across experiments. Group-level analysis revealed a main effect of Experiment, with post hoc comparisons showing that Experiment 2 produced stronger cueing effects than Experiments 1 and 3, whereas the latter two did not differ significantly (see Supplemental Material Sect. 6). The effect in Experiment 2 was particularly robust, emerging early and becoming more pronounced over time. Unlike Experiments 1 and 3, which relied on relatively local features—specific identities or precise facing directions—Experiment 2 was based on the ensemble emotion of the display. Participants may therefore have relied less on individual identities or expressions and instead processed the overall affective tone, a form of ensemble coding known to occur rapidly and automatically via parallel extraction (Chong & Treisman, 2003, 2005). Importantly, the emotional ratio functioned as a low-variance summary statistic of the display, compressing item-level variability into a stable aggregate signal. Prior work has shown that global non-social features can serve as contextual cues, although their effects are often modest under implicit learning conditions (Kunar et al., 2006). When spatial information is highly noisy or non-diagnostic, as in the present design with randomized layouts and positional jitter, learning mechanisms may preferentially weight cues that provide stable structure across the display. In this sense, ensemble-level information may possess a computational advantage over local identity-based cues, as it does not depend on precise item-by-item binding and remains relatively stable despite spatial perturbations.

This pattern is particularly informative in light of ongoing debates regarding the reproducibility of object-based contextual cueing (Makovski, 2016; Sisk et al., 2019). Several studies have reported difficulty reproducing object-based effects when spatial regularities are eliminated. For example, Gaál et al. (2026) did not observe a reliable cueing effect at the group level when spatial layouts were randomized, raising concerns that object-based learning may be inherently fragile. A key methodological difference between Gaál et al. (2026) and the present Experiment 1 concerns the degree of distractor overlap across mapping conditions. In Gaál et al. (2026), the same pool of distractor identities was used to construct both repeated and new configurations, such that distractor identities were shared across mapping conditions. This overlap may have reduced the extent to which object identity provided uniquely diagnostic information for signaling contextual repetition. In contrast, following Chun and Jiang (1999), the present Experiment 1 assigned distractor identities to two non-overlapping pools, one for each mapping condition, such that distractors appearing in Consistent Mapping trials were never reused in Variable Mapping trials. Under these conditions, object identity provided uniquely diagnostic information for distinguishing repeated from non-repeated contexts, thereby enabling reliable contextual learning despite the absence of spatial regularities. These findings suggest that distractor overlap across mapping conditions may be a critical determinant of whether object-based contextual cueing reliably emerges. In particular, inconsistencies across studies may reflect differences in the degree to which available object information forms a stable and uniquely diagnostic relational context. Under such conditions, social relational structure may provide a predictive scaffold that stabilizes contextual learning even in the absence of spatial consistency.

A similar logic applies to Experiment 2 under more constrained conditions. In this experiment, spatial information remained non-diagnostic, and identity information (excluding the target) was held constant across mapping conditions, minimizing competition from local or item-specific cues. As a result, the global emotional composition of the display constituted the most informative dimension available for distinguishing Consistent and Variable Mapping trials. Because this ensemble cue was both stable and uniquely diagnostic, it did not need to compete with partially informative alternatives. In addition, ensemble emotion can be extracted rapidly as a global summary statistic, which may have provided a particularly efficient signal for contextual learning. Importantly, these findings do not imply that object-based contextual cueing is generally robust, nor do they specify its underlying mechanism. Rather, they suggest that contextual learning effects are more likely to emerge reliably at the group level when a socially meaningful cue is simultaneously stable, uniquely diagnostic, and unconfounded by competing sources of information.

Nonetheless, a potential concern is whether the contextual cueing observed in Experiment 2 was driven by high-level ensemble emotion or by low-level visual regularities. As noted in prior research, attentional guidance for emotional faces can be influenced by salient low-level features, such as teeth visibility, which often differs systematically between happy and angry expressions (Becker et al., 2011; Horstmann et al., 2012). Because the stimuli consisted of happy and angry faces, we examined teeth visibility as one representative example of a low-level visual feature associated with emotional expression. Using a brightness-based pixel proxy (see Supplemental Material Sect. 3), we found that teeth visibility showed only a negligible association with search performance, did not differ between Consistent Mapping and Variable Mapping conditions, and did not modulate the Mapping effect when statistically controlled in a regression model. Importantly, even after statistically controlling for this low-level proxy, the Mapping effect remained robust and highly significant. Thus, the contextual cueing effect cannot be readily attributed to pixel-level brightness differences.

From a theoretical perspective, social emotions are perceived through the integration of multiple diagnostic cues (e.g., mouth curvature, eye wrinkles), and ensemble perception relies on the statistical summary of such features rather than any single visual component. Given this holistic nature of emotion perception, isolating a single low-level feature from the broader social construct is both theoretically and practically difficult. A related consideration applies to Experiment 3. Although the white background regions surrounding the faces could, in principle, introduce low-level visual differences across facing and non-facing configurations, our quantitative analysis of global luminance statistics demonstrated that display-level brightness distributions were nearly identical across Mapping conditions (see Supplemental Material Sect. 4). Internal luminance similarity was extremely high in both Consistent and Variable Mapping conditions, and the difference between conditions was less than.001. These results indicate that global pixel-level visual statistics were tightly matched across conditions and are unlikely to have served as stable predictive cues. Taken together, this pattern of findings suggests that the contextual cueing effect in Experiment 3 is better explained by learning of relational social orientation information rather than by sensitivity to low-level brightness patterns.

Regarding the overall magnitude of the effects across experiments, the contextual cueing effect in Experiments 1 and 3 was reliable but smaller than in Experiment 2. First, in Experiment 1, several factors may have limited the strength of the identity-based cue. The participants viewed a large number of other-race faces, and the well-known other-race effect (Malpass & Kravitz, 1969) may have reduced the discriminability of individual identities. Additionally, the stimulus set was much larger than in the other experiments: 96 unique faces were shown, with 48 appearing in the Consistent Mapping condition, compared with 15 and 14 unique faces in Experiments 2 and 3, respectively. The cognitive load associated with learning identity pairings across so many items may have weakened the contextual cueing effect.

Experiment 3 involved relational information—specifically, who was facing whom within face pairs. Relational cues require integrating directional information across multiple elements and thus may impose greater representational demands than either local identity pairings or global emotional composition. Nevertheless, the reliable learning observed in Experiment 3 demonstrates that people can implicitly encode and use relational social structure even when it requires integrating information across several pairs.

The awareness results across experiments suggest that learning was primarily implicit. Although some participants reported noticing regularities, particularly in Experiment 2, their ability to reproduce those regularities in the forced-choice generation task was limited. This dissociation between subjective reports and objective performance aligns with ongoing debates about the role of explicit awareness in contextual learning (e.g., Luque et al., 2017; Vadillo et al., 2016, 2020). Subjective recognition reports may overestimate awareness by capturing vague intuitions or post hoc impressions, whereas performance-based measures such as forced-choice generation may underestimate partial or fragmentary knowledge. Relatedly, poor performance in awareness tasks does not necessarily imply a complete absence of awareness, as it may reflect memory decay or weakened representations rather than a lack of initial learning.

To address these concerns, we conducted additional analyses reported in the Supplemental Materials (Sect. 5), directly examining whether awareness measures were functionally related to contextual cueing across all three experiments. These analyses tested the relationship between both subjective recognition reports and objective generation performance and the magnitude of contextual cueing. Across experiments, neither subjective awareness nor objective explicit knowledge reliably predicted contextual cueing, and participants who reported noticing regularities did not show greater cueing than those who did not. Although these measures cannot fully resolve broader theoretical debates about awareness in learning, the consistent pattern across experiments suggests that explicit knowledge was not systematically related to contextual cueing and does not appear to have been functionally necessary for its emergence in the present experiments.

The present findings have implications for broader theories of social cognition and learning. Social environments are rich with recurring patterns—such as who interacts with whom, how groups express collective emotion, and how individuals orient themselves relative to others. Efficient navigation of these environments may depend on the ability to implicitly detect and integrate such regularities. The present results suggest that contextual learning mechanisms can incorporate socially meaningful information, potentially providing a preparatory foundation for interpreting and predicting social interactions. Moreover, social cues are known to be detected rapidly and automatically, while remaining accessible for conscious evaluation (Adolphs, 2009), reflecting their central role in survival, communication, and cultural interaction (Arioli et al., 2018). Consistent with theories proposing specialized neural pathways for social information (Pitcher & Ungerleider, 2021), the present findings indicate that socially meaningful cues can be integrated into predictive learning mechanisms even when they are not defined by spatial regularities. In this sense, the ability to implicitly learn and exploit social regularities may reflect evolved cognitive and social mechanisms that enable humans to navigate complex social environments efficiently.

Several limitations should be acknowledged. The stimuli were static and simplified, and the social cues were isolated rather than embedded in complex, dynamic environments. Real-world social scenes contain multiple overlapping cues—such as identity, emotion, gaze, posture, and movement—whose relevance can change over time. Future work should therefore investigate how multiple social cues interact, how their relative influence shifts across contexts, and how individual differences in traits such as social anxiety, empathy, loneliness, or cultural background modulate implicit learning of social structure. Consistent with this possibility, prior work has shown that cultural background influences ensemble perception of faces (Im et al., 2017; Son et al., 2023) and that social anxiety can bias the recognition of ensemble emotion (Yang et al., 2013). Finally, the use of dynamic stimuli—such as videos of social interactions or immersive virtual environments—may provide more ecologically valid tests of these processes.

In addition, the present study cannot determine the processing stage at which the contextual cueing effect operates. It remains possible that the observed facilitation primarily reflects post-search or decisional processes rather than early attentional guidance (Kunar et al., 2007). Nevertheless, recent ERP evidence—specifically modulations of the N2pc and P3b components—suggests that contextual cueing can influence both early attentional and later decision-related processes (Nagy et al., 2024), leading us to suggest that contextual learning is likely supported by contributions from multiple processing stages rather than a single, isolated locus. Finally, clarifying the precise mechanisms and boundary conditions of object-based contextual cueing remains an important goal for future research.

In conclusion, the present study provides converging evidence that social information can function as a contextual cue in visual search even in the absence of spatial regularities. Across diverse social cue types, participants implicitly learned stable mappings and used them to guide attention. Rather than suggesting that object-based contextual cueing robustly generalizes beyond spatial structure, these findings indicate that socially meaningful relational structure may provide a predictive scaffold that stabilizes contextual learning when spatial consistency is absent. In this sense, the flexibility of contextual learning mechanisms may depend not simply on repetition, but on whether repeated elements form coherent and diagnostically informative relational structures. Future research using more complex, naturalistic stimuli will further clarify how social information is prioritized, integrated, and learned in real-world environments.

## Supplementary Information

Below is the link to the electronic supplementary material.

Supplementary file1 (DOCX 472 KB)

## Data Availability

All datasets and analysis scripts associated with the experiments are available on the Open Science Framework (https://osf.io/etz9w/?view_only=19963df218d041be8e3e781df99226b1). The face stimulus materials cannot be made publicly available because they were developed by other researchers and are distributed under restricted access with permission from the copyright holders. The study was not preregistered.

## Abbreviations

BF: Bayes factor
RaFD: Radboud faces database
ms: Milliseconds
RT: Reaction time
SD: Standard deviation
BF_incl_: Inclusion Bayes factor

## References

[CR1] Abramson, L., Petranker, R., Marom, I., & Aviezer, H. (2021). Social interaction context shapes emotion recognition through body language, not facial expressions. *Emotion,**21*(3), 557. 10.1037/emo0000718 31971411

[CR2] Adolphs, R. (2009). The social brain: Neural basis of social knowledge. *Annual Review of Psychology,**60*(1), 693–716. 10.1146/annurev.psych.60.110707.163514 18771388 PMC2588649

[CR3] Appelbaum, M., Cooper, H., Kline, R. B., Mayo-Wilson, E., Nezu, A. M., & Rao, S. M. (2018). Journal article reporting standards for quantitative research in psychology: The APA publications and communications board task force report. *American Psychologist,**73*(1), 3. 29345484 10.1037/amp0000191

[CR4] Arioli, M., Crespi, C., & Canessa, N. (2018). Social cognition through the lens of cognitive and clinical neuroscience. *BioMed Research International,**2018*(1), Article 4283427. 10.1155/2018/4283427 30302338 PMC6158937

[CR5] Becker, S. I., Horstmann, G., & Remington, R. W. (2011). Perceptual grouping, not emotion, accounts for search asymmetries with schematic faces. *Journal of Experimental Psychology: Human Perception and Performance,**37*(6), 1739. 10.1037/a0024665 21806311

[CR6] Brockmole, J. R., & Henderson, J. M. (2006). Using real-world scenes as contextual cues for search. *Visual Cognition,**13*(1), 99–108. 10.1080/13506280500165188

[CR7] Brockmole, J. R., Castelhano, M. S., & Henderson, J. M. (2006). Contextual cueing in naturalistic scenes: Global and local contexts. *Journal of Experimental Psychology: Learning, Memory, and Cognition,**32*(4), Article 699. 10.1037/0278-7393.32.4.699 16822141

[CR8] Chen, X., Bai, S., Ren, Q., Chen, Y., Long, F., & Jiang, Y. (2024). The representation of contextual cue is stimulus-specific yet its expression is flexible. *PeerJ,**12*, Article e17318. 10.7717/peerj.17318 38708357 PMC11067901

[CR9] Chong, S. C., & Treisman, A. (2003). Representation of statistical properties. *Vision Research,**43*(4), 393–404. 10.1016/S0042-6989(02)00596-5 12535996

[CR10] Chong, S. C., & Treisman, A. (2005). Statistical processing: Computing the average size in perceptual groups. *Vision Research,**45*(7), 891–900. 10.1016/j.visres.2004.10.004 15644229

[CR11] Chun, M. M., & Jiang, Y. (1998). Contextual cueing: Implicit learning and memory of visual context guides spatial attention. *Cognitive Psychology,**36*(1), 28–71. 10.1006/cogp.1998.0681 9679076

[CR12] Chun, M. M., & Jiang, Y. (1999). Top-down attentional guidance based on implicit learning of visual covariation. *Psychological Science,**10*(4), 360–365. 10.1111/1467-9280.00168

[CR13] de Fockert, J., & Wolfenstein, C. (2009). Short article: Rapid extraction of mean identity from sets of faces. *Quarterly Journal of Experimental Psychology,**62*(9), 1716–1722. 10.1080/17470210902811249 19382009

[CR14] Ding, X., Gao, Z., & Shen, M. (2017). Two equals one: Two human actions during social interaction are grouped as one unit in working memory. *Psychological Science,**28*(9), 1311–1320. 10.1177/0956797617707318 28719763

[CR15] Fu, Y., Zhou, M., Zhou, J., Shen, M., & Chen, H. (2024). Unconscious prioritization for face-to-face people. *Journal of Experimental Psychology: General,**153*(5), Article 1268. 38647479 10.1037/xge0001556

[CR16] Gaál, Z. A., Kojouharova, P., Czigler, I., & Nagy, B. (2026). Revisiting object contextual cueing: A replication study on implicit learning. *Acta Psychologica,**263*, Article 106214. 10.1016/j.actpsy.2026.106214 41518880

[CR17] Goujon, A., Didierjean, A., & Marmeche, E. (2009). Semantic contextual cuing and visual attention. *Journal of Experimental Psychology: Human Perception and Performance,**35*(1), 50. 10.1037/0096-1523.35.1.50 19170470

[CR18] Gray, K. L., Barber, L., Murphy, J., & Cook, R. (2017). Social interaction contexts bias the perceived expressions of interactants. *Emotion,**17*(4), 567. 10.1037/emo0000257 28191995

[CR19] Haberman, J., & Whitney, D. (2007). Rapid extraction of mean emotion and gender from sets of faces. *Current Biology,**17*(17), R751–R753. 10.1016/j.cub.2007.06.039 17803921 PMC3849410

[CR20] Haberman, J., & Whitney, D. (2009). Seeing the mean: Ensemble coding for sets of faces. *Journal of Experimental Psychology: Human Perception and Performance,**35*(3), 718. 10.1037/a0013899 19485687 PMC2696629

[CR21] Horstmann, G., Lipp, O. V., & Becker, S. I. (2012). Of toothy grins and angry snarls—Open mouth displays contribute to efficiency gains in search for emotional faces. *Journal of Vision,**12*(5), 7–7. 10.1167/12.5.7 22637708

[CR22] Im, H. Y., Albohn, D. N., Steiner, T. G., Cushing, C. A., Adams, R. B., Jr., & Kveraga, K. (2017). Differential hemispheric and visual stream contributions to ensemble coding of crowd emotion. *Nature Human Behaviour,**1*(11), 828–842. 10.1038/s41562-017-0225-z 29226255 PMC5716353

[CR23] JASP Team (2024). JASP (Version 0.19.2)[Computer software]

[CR24] Jeffreys, H. (1961). *Theory of probability* (3rd ed.). Oxford University Press.

[CR25] Kawahara, J. I. (2007). Auditory-visual contextual cuing effect. *Perception & Psychophysics,**69*, 1399–1408. 10.3758/BF03192955 18078230

[CR26] Kunar, M. A., Flusberg, S. J., & Wolfe, J. M. (2006). Contextual cuing by global features. *Perception & Psychophysics,**68*(7), 1204–1216. 10.3758/BF03193721 17355043 PMC2678916

[CR27] Kunar, M. A., Flusberg, S., Horowitz, T. S., & Wolfe, J. M. (2007). Does contextual cuing guide the deployment of attention? *Journal of Experimental Psychology: Human Perception and Performance,**33*(4), 816. 10.1037/0096-1523.33.4.816 17683230 PMC2922990

[CR28] Langner, O., Dotsch, R., Bijlstra, G., Wigboldus, D. H., Hawk, S. T., & Van Knippenberg, A. D. (2010). Presentation and validation of the Radboud Faces Database. *Cognition and Emotion,**24*(8), 1377–1388. 10.1080/02699930903485076

[CR29] Lee, M. D., & Wagenmakers, E.-J. (2013). *Bayesian Modeling for Cognitive Science: A Practical Course*. Cambridge University Press

[CR30] Luque, D., Vadillo, M. A., Lopez, F. J., Alonso, R., & Shanks, D. R. (2017). Testing the controllability of contextual cuing of visual search. *Scientific Reports,**7*, Article 39645. 10.1038/srep39645 28045108 PMC5206715

[CR31] Makovski, T. (2016). What is the context of contextual cueing? *Psychonomic Bulletin & Review,**23*, 1982–1988. 10.3758/s13423-016-1058-x 27215750

[CR32] Makovski, T. (2018). Meaning in learning: Contextual cueing relies on objects’ visual features and not on objects’ meaning. *Memory & Cognition,**46*, 58–67. 10.3758/s13421-017-0745-9 28770539

[CR33] Malpass, R. S., & Kravitz, J. (1969). Recognition for faces of own and other race. *Journal of Personality and Social Psychology,**13*(4), 330. 10.1037/h0028434 5359231

[CR34] Minear, M., & Park, D. C. (2004). A lifespan database of adult facial stimuli. *Behavior Research Methods, Instruments, & Computers,**36*, 630–633. 10.3758/BF03206543 15641408

[CR35] Nagy, B., Kojouharova, P., Protzner, A. B., & Gaál, Z. A. (2024). Investigating the effect of contextual cueing with face stimuli on electrophysiological measures in younger and older adults. *Journal of Cognitive Neuroscience,**36*(5), 776–799. 10.1162/jocn_a_02135 38437174

[CR36] Paparella, I., & Papeo, L. (2022). Chunking by social relationship in working memory. *Visual Cognition,**30*(5), 354–370. 10.1080/13506285.2022.2064950

[CR37] Peirce, J., Gray, J. R., Simpson, S., MacAskill, M., Höchenberger, R., Sogo, H., Kastman, E., & Lindeløv, J. K. (2019). PsychoPy2: Experiments in behavior made easy. *Behavior Research Methods,**51*(1), 195–203. 10.3758/s13428-018-01193-y 30734206 PMC6420413

[CR38] Pitcher, D., & Ungerleider, L. G. (2021). Evidence for a third visual pathway specialized for social perception. *Trends in Cognitive Sciences,**25*(2), 100–110. 10.1016/j.tics.2020.11.006 33334693 PMC7811363

[CR39] Rouder, J. N. (2014). Optional stopping: No problem for Bayesians. *Psychonomic Bulletin & Review,**21*, 301–308. 10.3758/s13423-014-0595-4 24659049

[CR40] RStudio Team. (2020). *RStudio: Integrated development for R*. RStudio, PBC.

[CR41] Sisk, C. A., Remington, R. W., & Jiang, Y. V. (2019). Mechanisms of contextual cueing: A tutorial review. *Attention, Perception, & Psychophysics,**81*, 2571–2589. 10.3758/s13414-019-01832-2 31410759

[CR42] Skripkauskaite, S., Mihai, I., & Koldewyn, K. (2023). Attentional bias towards social interactions during viewing of naturalistic scenes. *Quarterly Journal of Experimental Psychology,**76*(10), 2303–2311. 10.1177/17470218221140879 PMC10503253 36377819

[CR43] Son, G., Im, H. Y., Albohn, D. N., Kveraga, K., Adams, R. B., Jr., Sun, J., & Chong, S. C. (2023). Americans weigh an attended emotion more than Koreans in overall mood judgments. *Scientific Reports,**13*(1), Article 19323. 10.1038/s41598-023-46723-7 37935828 PMC10630378

[CR44] Tavera, F., & Haider, H. (2025). The role of selective attention in implicit learning: Evidence for a contextual cueing effect of task-irrelevant features. *Psychological Research Psychologische Forschung,**89*(1), 15. 10.1007/s00426-024-02033-9 PMC11564302 39540996

[CR45] Vadillo, M. A., Konstantinidis, E., & Shanks, D. R. (2016). Underpowered samples, false negatives, and unconscious learning. *Psychonomic Bulletin & Review,**23*(1), 87–102. 10.3758/s13423-015-0892-6 26122896 PMC4742512

[CR46] Vadillo, M. A., Linssen, D., Orgaz, C., Parsons, S., & Shanks, D. R. (2020). Unconscious or underpowered? Probabilistic cuing of visual attention. *Journal of Experimental Psychology: General,**149*(1), 160. 10.1037/xge0000632 31246061

[CR47] Vestner, T., Tipper, S. P., Hartley, T., Over, H., & Rueschemeyer, S. A. (2019). Bound together: Social binding leads to faster processing, spatial distortion, and enhanced memory of interacting partners. *Journal of Experimental Psychology: General,**148*(7), 1251. 10.1037/xge0000545 30652892

[CR48] Yang, J. W., Yoon, K. L., Chong, S. C., & Oh, K. J. (2013). Accurate but pathological: Social anxiety and ensemble coding of emotion. *Cognitive Therapy and Research,**37*, 572–578. 10.1007/s10608-012-9500-5

