# Supplementary material for: Implicit learning of social information in contextual cueing
Source: Cogn Res Princ Implic. 2026 Jun 23;11:48. doi: 10.1186/s41235-026-00736-8 (PMC13547194; doi:10.1186/s41235-026-00736-8)
Supplement: Supplementary file 1 [file 41235_2026_736_MOESM1_ESM.docx]

**Supplemental Materials**

**Implicit Learning of Social Information in Contextual Cueing**

**Contents**

**1) Femininity Rating Procedure for Target Counterbalancing**

To minimize potential confounds of perceived femininity on task performance, all 16 female faces used as target stimuli in Experiment 1 were evaluated in a separate rating task. An independent group of 10 participants (5 men, 5 women) rated each face on a 7-point Likert scale (1 = not at all feminine, 7 = very feminine). The mean rating for each face was calculated and used to balance femininity levels across mapping conditions. Specifically, the 16 faces were ranked by average femininity rating and split into two groups of odd- and even-ranked faces. For counterbalancing, half of the participants received the odd-numbered faces in the consistent mapping condition and the even-numbered faces in the variable mapping condition, while the assignment was reversed for the other half. This procedure ensured that participants were exposed to target faces with comparable levels of perceived femininity across conditions, preventing potential bias from gendered facial features. Overall, the faces had a moderate level of perceived femininity (*M* = 4.41, *SD* = 1.77), with substantial variability across stimuli.

**Figure S1**

*Mean femininity ratings for each of the 16 female faces used in Experiment 1.*

*Note*. Error bars represent standard errors of the mean.

**2) Results Across Overall Mood Ratio in Experiment 2**

To examine whether reaction times differed depending on the overall mood ratio, we conducted an additional analysis based on counterbalanced group assignments. Both groups were exposed to 14 different Angry:Happy ratios, ranging from 14:1 to 1:14. For Group 1, the consistent mapping condition included ratios in which the number of Angry faces was even (e.g., 14:1, 12:3, 10:5). In contrast, for Group 2, the consistent mapping condition included ratios in which the number of Angry faces was odd (e.g., 13:2, 11:4, 9:6).

We conducted a Bayesian repeated measures ANOVA with mapping condition and overall mood ratio as within-subject factors and group as a between-subject factor. The best-fitting model included the main effects of mapping and group, as well as their interaction, BF_10_ = 4.28 × 10^5^. There was extremely strong evidence for the main effect of mapping (BF_incl_ = 1.62 × 10^5^), with participants responding on average 0.17 s faster in the consistent mapping condition than in the variable condition. The Mapping × Group interaction showed moderate evidence (BF_incl_ = 5.41). In contrast, the main effect of overall mood ratio (BF_incl_ = 0.19) and all remaining effects yielded evidence against including these terms, suggesting that they did not contribute meaningfully to the model. The overall pattern of reaction times is presented in Figure S2.

**Figure S2**

*Response times across overall mood ratios by group in Experiment 2.*

*Note*. (a) Group 1 and (b) Group 2: Response times across overall mood ratios (angry faces : happy faces) for the two mapping conditions. Error bars represent standard errors of the mean.

**3) Additional Analyses of Low-Level Visual Features (Teeth Visibility) in Experiment 2**

To evaluate whether low-level visual features associated with emotional expressions could have contributed to the contextual cueing effect observed in Experiment 2, we conducted additional control analyses. Emotional facial expressions vary in both affective meaning and in certain salient visual properties. Prior research has shown that attentional guidance can be influenced by low-level features such as teeth visibility, which tends to differ systematically between happy and angry expressions (Becker et al., 2011; Horstmann et al., 2012).

Because the stimulus set in Experiment 2 consisted of happy and angry faces, we examined whether visually salient features that typically distinguish these expressions might have influenced performance. Among these features, teeth visibility represents one of the most salient and quantifiable low-level visual differences between happy and angry expressions. Happy faces more frequently involve exposed teeth, whereas angry faces typically do not. Accordingly, as a representative example of a low-level visual feature associated with emotional expression, we focused on teeth visibility and evaluated whether it could account for the observed contextual cueing effect.

Teeth visibility was operationalized using a brightness-based pixel proxy implemented in Python. Each facial image was converted to grayscale, and a rectangular region of interest (ROI) corresponding to the lower central portion of the face was defined. Within this ROI, pixels exceeding a predefined brightness threshold were counted and used as an estimate of teeth visibility. This procedure did not involve explicit segmentation or classification of teeth, but instead provided a coarse stimulus-driven proxy for relative brightness in the mouth region.

To comprehensively assess the potential role of this low-level feature, three analyses were conducted sequentially: (a) a trial-level correlation analysis between teeth visibility and RT, (b) a test of equivalence of teeth pixel counts across mapping conditions, and (c) a regression analysis examining whether the mapping effect remained after statistically controlling for teeth visibility.

(a) **Trial-level correlation between teeth visibility and RT**

For each trial of the visual search task, total teeth pixel count was computed by summing the estimated teeth pixels across all faces presented on the screen, including both the target and all distractors. Trials with incorrect responses were excluded from this analysis. Across all participants and trials (*N* = 8,839), a small positive correlation was observed between total teeth pixel count and reaction time, *r* = .041, *p* < .001, accounting for less than 0.2% of the variance (R² = .0017). Notably, greater overall teeth visibility was associated with slightly slower responses. Although statistically reliable due to the large number of trials, the magnitude of this effect was negligible.

(b) **Condition-level differences in teeth visibility**

We next examined whether total teeth pixel counts differed systematically between the Consistent Mapping condition and the Variable Mapping condition. Across all trials, the mean total teeth pixel count was comparable between the two conditions (Consistent Mapping: *M* = 288.41, *SD* = 136.43; Variable Mapping: *M* = 289.08, *SD* = 136.98). An independent-samples t-test revealed no significant difference between conditions, *t*(8837) = −0.23, *p* = .819, BF_10_ = 0.025.

Separate analyses conducted within each emotion yielded similar results. In Angry trials, teeth pixel counts did not differ between the Consistent Mapping condition (*M* = 279.97, *SD* = 135.21) and the Variable Mapping condition (*M* = 281.56, *SD* = 135.80), *t*(4398.09) = −0.38, *p* = .703, BF_10_ = 0.036. In Happy trials, the Consistent Mapping condition (*M* = 296.75, *SD* = 137.42) and the Variable Mapping condition (*M* = 296.56, *SD* = 138.01) likewise showed no significant difference, *t*(4434) = 0.05, *p* = .962, BF_10_ = 0.034.

In addition, examination of the stimulus set indicated that not all happy-face stimuli displayed visible teeth, as several happy faces yielded a teeth pixel count of zero under the present operationalization. This observation indicates that emotion and teeth visibility were not in a one-to-one correspondence in the stimuli used, further reducing the likelihood that emotional structure was confounded with pixel-level brightness differences. Taken together, these findings suggest that the repeated Mapping conditions were not associated with systematic differences in the brightness-based proxy of teeth visibility.

(c) **Effect-level analysis controlling for teeth visibility**

To determine whether low-level brightness-based features could account for the contextual cueing effect, we conducted a multiple regression analysis using a full-factorial regression model (RT ~ Mapping × Emotion × Teeth Pixel). Crucially, even after statistically controlling for teeth visibility and emotion, the main effect of Mapping condition remained highly significant, *b* = 0.395, *SE* = 0.073, *t* = 5.39, *p* < .001. This indicates that the contextual cueing effect persisted independently of variation in the brightness-based proxy of teeth visibility.

Importantly, neither the Mapping × Teeth Pixel interaction (*p* = .67) nor the Mapping × Emotion × Teeth Pixel interaction (*p* = .68) was significant. These results indicate that the magnitude of the Mapping effect did not vary as a function of teeth visibility. In other words, pixel-level brightness differences in the mouth region did not modulate the contextual cueing effect. Taken together, these findings suggest that the Mapping effect observed in Experiment 2 cannot be attributed to low-level brightness-based features associated with emotional expressions.

Across trial-level, condition-level, and effect-level analyses, no evidence was found that brightness-based estimates of teeth visibility accounted for the contextual cueing effect observed in Experiment 2. Teeth visibility showed only a negligible association with RT, did not differ systematically across Mapping conditions, and did not moderate the Mapping effect when statistically controlled. Collectively, these results indicate that the contextual cueing effect cannot be explained by low-level visual features associated with emotional expressions and is therefore unlikely to reflect learning of pixel-level brightness differences.

**4) Additional Analysis of Low-Level Visual Statistics in Experiment 3**

To evaluate whether the contextual cueing effect observed in Experiment 3 could have been influenced by low-level visual regularities rather than relational facing-direction information, we conducted an additional quantitative analysis of global luminance statistics. The face stimuli used in the experiment contained white background regions outside the facial area due to characteristics of the stimulus dataset, and the experimental display background was gray. As a result, these white background regions may have been perceptually salient, and their overall shape or distribution could have differed systematically between facing and non-facing configurations. If such low-level differences were present and stable across Mapping conditions, they could in principle contribute to attentional guidance independently of relational social structure.

All stimulus images were converted to grayscale, and a 256-bin global intensity histogram (0–255) was computed for each image to characterize the distribution of pixel brightness values. The original display configurations were reconstructed using the orientation variables recorded in the raw data. For each trial, the histograms of all 14 faces presented on the screen (one target and thirteen distractors) were aggregated and averaged to generate a display-level luminance profile. To minimize incidental biases arising from specific identity pairings, each trial was reconstructed 10 times with randomly sampled face identities, and the resulting luminance profiles were averaged. Pearson correlations were then computed between the 256-dimensional histogram vectors across trials within each Mapping condition to quantify internal physical similarity.

Across all 20 participants, internal luminance similarity was extremely high in both Mapping conditions (Consistent Mapping: *r* = .999; Variable Mapping: *r* = .999). Although a statistical comparison yielded a significant t-value (*t* = −326.38, *p* < .001), this result reflected the very large number of sampled comparisons; the difference between conditions was less than .001. Thus, the global luminance distributions of displays in the two Mapping conditions were nearly identical.

These findings indicate that facing and non-facing configurations did not produce meaningful differences in global brightness statistics at the display level. The extremely high internal similarity observed in both Mapping conditions suggests that low-level luminance structure was tightly controlled and highly comparable across trials. Therefore, it is unlikely that the contextual cueing effect observed in Experiment 3 can be attributed to global pixel-level visual regularities. Instead, the results are more consistent with learning of relational social orientation information rather than sensitivity to low-level brightness patterns.

**5) Additional Analysis on the Relationship between Awareness and Learning**

To further evaluate whether explicit knowledge contributed to the observed contextual cueing effects, we conducted a series of additional analyses examining the relationship between awareness and learning across all three experiments. Awareness was assessed using two measures administered after the visual search task: first, a recognition test assessing subjective reports of noticing regularities, and second, a forced-choice generation task assessing objective knowledge via accuracy. Learning was indexed by the magnitude of the contextual cueing effect (CCE) during the Late Phase of the visual search task. For Experiments 2 and 3, three complementary analyses were performed: (1) correlation analyses between forced-choice generation accuracy and CCE magnitude, (2) comparisons of CCE magnitude between participants whose forced-choice performance was above chance and those whose performance was at or below chance, and (3) comparisons of CCE magnitude between participants who reported noticing regularities and those who did not. For Experiment 1, only the first two analyses were conducted because no participants reported noticing any regularities in the recognition test, which precluded meaningful comparisons based on subjective awareness.

**5.1. Experiment 1**

In Experiment 1, participants learned associations between target identities and distractor identities. No participants subjectively reported noticing regularities in the recognition test, so the following analyses focus exclusively on objective knowledge measures.

*Correlation between Explicit Knowledge and CCE.*

We first examined whether accuracy in the forced-choice generation task was related to the magnitude of the CCE during the Late Phase. No significant correlation was observed (*r* = −.11, *p* = .66), and Bayesian correlation analysis provided anecdotal evidence in favor of the null hypothesis (BF

_10_ =0.51).

*Comparison by Explicit Knowledge Groups.*

Participants were divided into an Above-Chance group (*n* = 5) and a Below-Chance group (*n* = 15) based on the chance level of 12.5% in the forced-choice generation task. A Bayesian independent-samples *t*-test revealed no reliable difference in CCE magnitude between the two groups, *t*(18) = 0.51, *p* = .62, BF_10_ = 0.48. The mean CCE for the Above-Chance group (*M* = 0.107 s) was comparable to that of the Below-Chance group (*M* = 0.068 s), indicating that search facilitation remained invariant regardless of whether participants possessed detectable explicit knowledge.

**5.2. Experiment 2**

In Experiment 2, participants learned associations between the overall mood ratio of the display and target identity. The following analyses were conducted.

*Correlation between Explicit Knowledge and CCE.*

No significant correlation was observed between forced-choice generation accuracy and CCE magnitude during the Late Phase (*r* = .01, *p* = .96, BF_10_ = 0.52).

*Comparison by Explicit Knowledge Groups.*

Participants whose forced-choice performance was above chance (*n* = 5) did not differ reliably in CCE magnitude from those whose performance was at or below chance (*n* = 11), *t*(14) = 0.05, *p* = .96, BF_10_ = 0.45. The mean CCE was nearly identical in the Above-Chance group (*M* = 0.541 s) and the Below-Chance group (*M* = 0.537 s), indicating that robust contextual cueing was observed even in participants with no detectable explicit knowledge.

*Comparison by Subjective Recognition Reports.*

We next compared CCE magnitudes between participants who reported noticing regularities in the recognition test (*n* = 9) and those who reported no such noticing (*n* = 7). The analysis revealed no significant difference between the two groups, *t*(14) = 0.17, *p* = .87, BF_10_ = 0.43. Thus, subjective awareness as assessed by the recognition test did not reliably predict the magnitude of contextual cueing.

**5.3. Experiment 3**

In Experiment 3, which involved learning relational configurations defined by facing direction, the same set of analyses was conducted. Below, we report the key results from each analysis.

*Correlation between Explicit Knowledge and CCE.*

No reliable association was observed between explicit knowledge, as indexed by accuracy in the forced-choice generation task, and the magnitude of the CCE during the Late Phase (*r* = .07, *p* = .76, BF_10_ = 0.49).

*Comparison by Explicit Knowledge Groups.*

Participants whose forced-choice performance was above chance (*n* = 10) did not differ reliably in CCE magnitude from those whose performance was at or below chance (*n* = 10), *t*(18) = 0.19, *p* = .85, BF_10_ = 0.40. Notably, participants in the below-chance group showed a CCE (*M* = 0.133 s) comparable in magnitude to that observed in the above-chance group (*M* = 0.142 s), indicating that contextual cueing was present even in the absence of detectable explicit knowledge.

*Comparison by Subjective Recognition Reports.*

Similarly, CCE magnitudes did not differ between participants who reported noticing regularities in the recognition test (*n* = 9) and those who reported no such noticing (*n* = 11), *t*(18) = −0.36, *p* = .72, BF_10_ = 0.42.

**5.4. Summary of Evidence for Implicit Learning**

In summary, the additional analyses across all experiments provide converging evidence that explicit knowledge was not systematically related to the magnitude of contextual cueing. Across multiple indices of awareness—including objective performance in the forced-choice generation task and subjective reports from the recognition test—no reliable association with contextual cueing was observed. In particular, participants who reported noticing regularities in the recognition test did not show greater cueing than those who reported no such noticing. Although we acknowledge the difficulty of completely ruling out partial or fragmentary explicit representations, the statistical evidence across experiments consistently favored the null hypothesis. Taken together, these findings suggest that explicit knowledge was not functionally necessary for the emergence of contextual cueing and that awareness measures were not systematically related to the magnitude of the effect.

**6) Comparison of Contextual Cueing Effects Across Experiments**

To compare the magnitude of contextual cueing effects across the three experiments employing different types of social information, we conducted an additional analysis treating Experiment as a between-subject factor. The dependent variable was the contextual cueing effect, calculated by subtracting response times in the consistent mapping condition from those in the variable mapping condition (i.e., Variable – Consistent). A Bayesian one-way ANOVA revealed extremely strong evidence for a main effect of Experiment (BF_10_ = 5.96e+8). Post hoc comparisons indicated that the contextual cueing effect in Experiment 2 (*M* = 0.35 s, *SD* = 0.14 s) was greater than in both Experiment 1 (*M* = 0.03 s, *SD* = 0.11 s; BF_10_ = 1.21e+6) and Experiment 3 (*M* = 0.08 s, *SD* = 0.08 s; BF_10_ = 640,780.76). In contrast, the difference between Experiments 1 and 3 was not supported by the data (BF_10_ = 0.88), providing anecdotal evidence in favor of the null hypothesis These patterns are illustrated in Figure S3.

**Figure S3**

*Contextual cueing effects across the three experiments*

*Note*. Violin plots depict the distribution of contextual cueing effects, calculated as the difference in response times between the variable and consistent mapping conditions (Variable – Consistent). Positive values indicate faster responses in the consistent mapping condition. White boxes within the violins represent the interquartile range, with the horizontal line indicating the median. Each experiment used a different type of social cue: identity (Experiment 1), overall mood (Experiment 2), and facing direction (Experiment 3).

**References**

Becker, S. I., Horstmann, G., & Remington, R. W. (2011). Perceptual grouping, not emotion, accounts for search asymmetries with schematic faces. *Journal of Experimental Psychology: Human Perception and Performance*, *37*(6), 1739-1757.

Horstmann, G., Lipp, O. V., & Becker, S. I. (2012). Of toothy grins and angry snarls—Open mouth displays contribute to efficiency gains in search for emotional faces. *Journal of Vision*, *12*(5):7, 1-15
